# Renal Perfusion, Oxygenation and Metabolism: The Role of Imaging

**DOI:** 10.3390/jcm12155141

**Published:** 2023-08-06

**Authors:** Johanna Päivärinta, Ioanna A. Anastasiou, Niina Koivuviita, Kanishka Sharma, Pirjo Nuutila, Ele Ferrannini, Anna Solini, Eleni Rebelos

**Affiliations:** 1 Department of Medicine, Division of Nephrology, Turku University Hospital, 20521 Turku, Finland; johanna.paivarinta@tyks.fi (J.P.); niina.koivuviita@tyks.fi (N.K.); 2 1st Department of Propaedeutic and Internal Medicine, Medical School, National and Kapodistrian University of Athens, Laiko General Hospital, 11527 Athens, Greece; anastasiouiwanna@gmail.com; 3 Department of Imaging, Infection, Immunity and Cardiovascular Disease, University of Sheffield, Sheffield S10 2TN, UK; kanishka.sharma@sheffield.ac.uk; 4 Turku PET Centre, 20521 Turku, Finland; pirjo.nuutila@utu.fi; 5 Department of Endocrinology, Turku University Hospital, 20521 Turku, Finland; 6 CNR, Institute of Clinical Physiology, 56124 Pisa, Italy; eleferrannini@gmail.com; 7 Department of Surgical, Medical, Molecular and Critical Area Pathology, University of Pisa, 56124 Pisa, Italy; anna.solini@unipi.it; 8 Department of Clinical and Experimental Medicine, University of Pisa, 56124 Pisa, Italy

**Keywords:** positron emission tomography, functional magnetic resonance imaging, renal perfusion, renal oxygenation, renal substrate uptake

## Abstract

Thanks to technical advances in the field of medical imaging, it is now possible to study key features of renal anatomy and physiology, but so far poorly explored due to the inherent difficulties in studying both the metabolism and vasculature of the human kidney. In this narrative review, we provide an overview of recent research findings on renal perfusion, oxygenation, and substrate uptake. Most studies evaluating renal perfusion with positron emission tomography (PET) have been performed in healthy controls, and specific target populations like obese individuals or patients with renovascular disease and chronic kidney disease (CKD) have rarely been assessed. Functional magnetic resonance (fMRI) has also been used to study renal perfusion in CKD patients, and recent studies have addressed the kidney hemodynamic effects of therapeutic agents such as glucagon-like receptor agonists (GLP-1RA) and sodium-glucose co-transporter 2 inhibitors (SGLT2-i) in an attempt to characterise the mechanisms leading to their nephroprotective effects. The few available studies on renal substrate uptake are discussed. In the near future, these imaging modalities will hopefully become widely available with researchers more acquainted with them, gaining insights into the complex renal pathophysiology in acute and chronic diseases.

## 1. Introduction

Chronic kidney disease (CKD) is a global health burden [1] associated with unfavorable clinical and economic effects [2]. Early diagnosis is fundamental for preventing and delaying the progression of CKD [2]. Hypertension and type 2 diabetes (T2D) are leading causes of CKD while obesity and dyslipidemia have also been associated with an increased risk of CKD [3,4,5,6]. In particular, diabetic kidney disease (DKD) develops in about 30% of patients with T2D, nowadays representing the leading cause of end-stage renal disease (ESRD), and accounting for approximately 50% of all cases [7].

According to the chronic hypoxia hypothesis, renal tissue hypoxia is a unifying factor in the progression of CKD irrespective of its cause [8]. Hypoxia is associated with inflammation, vasoconstriction, vascular rarefaction, and tissue damage [9]. Given that tissue hypoxia contributes to both CKD and acute kidney injury (AKI), understanding the causes of tissue hypoxia is of paramount clinical importance.

No more than a decade ago, inhibitors of the renin-angiotensin system (RAS), i.e., angiotensin-converting enzyme inhibitors (ACEi) or angiotensin II receptor blockers (ARB), were the only available agents that were able to reduce albuminuria. Numerous clinical trials (the Collaborative Study Group (CSG) Captopril [10], the Angiotensin II Antagonist Losartan (RENAAL) [11], the irbesartan Microalbuminuria Study (IRMA)-2 and the Irbesartan Diabetic Nephropathy Trial (IDNT) trials [12,13]) have shown the ability of these treatments to limit albuminuria and slow the glomerular filtration rate of (GFR) decline. According to a Cochrane systematic review in 2006, taking an ACE inhibitor or an ARB was associated with a statistically significant lower risk of developing ESRD compared to a placebo (relative risk (RR): 0.60; 95% CI: 0.39–0.93, respectively) and macroalbuminuria (RR: 0.45; 95% CI: 0.29–0.69) [14].

The introduction of sodium-glucose co-transporter 2 inhibitors (SGLT2-i) in clinical practice changed the treatment of both DKD and CKD in non-diabetic individuals. After attracting the attention of the medical community for their impressive reduction in mortality and cardiovascular events [15], subsequent analyses showed that SGLT2-i exerts marked renal protective effects. Dedicated kidney outcome randomised controlled trials (RCT) (CREDENCE, DAPA-CKD, and EMPA-KIDNEY) reported reduced rates in composite renal outcomes, which was defined as the doubling of serum creatinine, end-stage renal disease or renal death in patients treated with SGLT2-i [16,17,18].

GLP-1 RA have also been shown to reduce albuminuria. A recent meta-analysis of seven RCTs showed that their treatment with GLP-1 RA reduced the risk of a combined kidney outcome compared to a placebo (HR: 0.83; 95% CI: 0.78–0.89) [19].

Finally, finerenone, a new highly selective nonsteroidal mineralocorticoid receptor antagonist (MRA), has also yielded promising results in terms of renal protection and decreasing albuminuria in a dose-dependent manner in a cohort of patients with T2D and CKD treated with RAS blockage [20,21]. Moreover, the Figaro-DKD trial showed that the kidney composite outcome (ESRD, a sustained ≥57% decrease in eGFR from baseline for ≥4 weeks, or renal death) occurred less frequently in the finerenone group compared to the placebo (HR: 0.77; 95% CI: 0.60–0.99) [21]. Currently, at least one RCT evaluated whether the combination of finerenone with SGLT2-i had additive effects on renal outcomes [22] (ClinicalTrials.gov Identifier: NCT05254002). 

In parallel with the quick improvement in the medical “arsenal” for halting the progression of CKD, the last few years have seen important progress in the field of medical imaging, enabling the in vivo study of renal pathophysiology in humans. Moreover, a multidisciplinary European network (European Cooperation in Science and Technology Action, PARENCHIMA) has been created that aims to disseminate the clinical use of renal MRI biomarkers. The various available MRI techniques and their main clinical/research applications were described in the position paper of PARENCHIMA [23], along with their technical recommendations [24].

In this narrative review, we focus on studies assessing renal perfusion (arterial spin labelling (ASL), magnetic resonance imaging (MRI), positron emission tomography (PET), renal oxygenation (blood oxygenation level-dependent (BOLD) MRI) and metabolism (PET), a triad of physiologic determinants of kidney function, which have so far not been extensively investigated in humans. Special interest has also been given to studies where the effects of SGLT2-i on renal physiology have been assessed. Other imaging modalities are extensively described elsewhere [23,25,26].

### 1.1. Renal Perfusion

Despite comprising <1% of body weight, the kidneys receive a high blood flow (~1.2–1.4 L/min), which accounts for ~20% of the cardiac output in the resting adult in the face of a small total kidney mass (300 gr). The kidneys have a unique perfusion system with two separate capillary beds, while the renal cortex receives a large blood flow: only 10% of renal blood flow perfuses the renal medulla, with evidence that cortical and medullary circulations are independently regulated [27]. Even in healthy subjects, the medulla appears to operate under conditions of quasi-hypoxia. The coupling between perfusion, oxygenation, and metabolism is complex and not completely understood. A preclinical study in rabbits showed that renal tissue PO_2_ is independent of the renal blood flow (RBF) because the preglomerular diffusional shunting of oxygen from the arteries to veins decreases with decreasing RBF and vice versa [28].

### 1.2. Principles of MRI-Measurement of Renal Perfusion (ASL) and Oxygenation (BOLD)

A dynamic contrast-enhanced MRI uses gadolinium for radiocontrast, which is cleared almost completely through glomerular filtration. Thus, gadolinium does not remain in the blood circulation, thereby complicating the evaluation of kidney perfusion. Furthermore, the nephrogenic systemic fibrosis that is associated with gadolinium use in patients with CKD mandates the cautious rational use of gadolinium. Ultrasmall superparamagnetic iron oxide particles like ferumoxytol have been introduced as kidney-safe MRI contrast agents [29]. 

**Figure 1:**
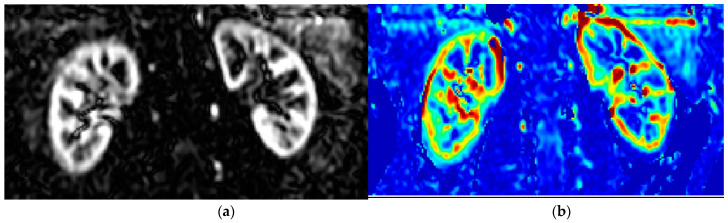
Representative perfusion weighted image (**a**) and the resulting RBF map (**b**) from the renal ASL (CASL) MRI technique on a healthy volunteer.

A fairly new MRI method to study renal perfusion is arterial spin labelling (ASL), which utilises radiofrequency (RF) pulses to alter the longitudinal magnetisation of water in the blood into a diffusible endogenous tracer [30]. A flow-sensitised “label” (or tag) image is acquired after a time delay to ensure the arrival of “labelled” blood to the renal tissue microvasculature. This image acquisition is then repeated to obtain a “control” image without altering the magnetisation of the inflowing blood. As the static tissue spins have the same magnetisation preparation; the difference in the signal of inflowing blood is only due to a different preparation, and subtracting the two resulting images yields a perfusion-weighted image (PWI) [31]. These PWIs are then fed into a model that describes the relationship between the difference in the signal and the actual blood perfusion. The result is a quantitative perfusion map (RBF) with units mL/100 g of tissue/min [31]. Different variants of ASL, such as continuous (CASL), pulsed (PASL), and pseudo-continuous (PCASL) [32,33], can be used to invert the arterial blood magnetization and obtain the renal tissue’s perfusion. Figure 1 shows an example of the ASL perfusion weighted image (left panel) and the resulting renal blood flow map (right panel) using the CASL technique.

BOLD imaging is another MRI sequence that allows the measurement of renal oxygenation. In principle, this sequence uses the paramagnetic properties of deoxyhemoglobin to assess tissue oxygenation. This measurement yields the relaxation rate R2*, which is proportional to deoxyhemoglobin levels: i.e., the higher the R2*, the lower the oxygenation. In a typical BOLD-fMRI study, the renal parenchyma is divided into 12 layers of equal thickness; the outer three layers are considered indicative of renal cortex oxygenation, whereas values from the 8th to 10th layer are representative of the medullary region. Other than evaluating the R2*, the slope of the linear part of the regression of the R2* values on the depth of the renal parenchyma (0% at the outer layer to 100% at the inner layer) can be used to express the oxygenation results [34].

### 1.3. Renal Perfusion and Renal Oxygenation Using MRI

Several studies have assessed renal perfusion using ASL [30] and oxygenation using BOLD MRI [35]. In an elegant study by Prasad and colleagues, patients with CKD and control subjects were studied with ASL, BOLD, and diffusion MRI. Renal cortical perfusion was reduced in CKD subjects compared to the controls (109.54 ± 25.38 vs. 203.17 ± 27.47 mL/min/100 g; *p* < 0.001); however, there was no difference in cortical oxygenation, nor were oxygenation or perfusion related to each other. However, as the authors discussed, it is unknown whether oxygenation is decreased in patients with CKD, as BOLD MRI might not be sufficiently sensitive to detect subtle deficits [36]. 

Zanchi et al. studied the acute and chronic effects of 10 mg empagliflozin in 45 healthy volunteers with eGFR > 60 mL/min/1.73 m^2^ and normoalbuminuria; in these healthy volunteers, SGLT2-i did not induce any acute or chronic effects on cortical and medullary oxygenation [37]. In a placebo-controlled cross-over trial, 15 patients with type 1 diabetes (T1D) received a single administration of dapagliflozin with 50 mg or a placebo; compared to the placebo, treatment with dapagliflozin improved renal cortical oxygenation, while it did not affect perfusion [38]. The authors suggested that this increase in cortical oxygenation could be attributed to a reduced tubular transport workload in the proximal tubules. The eventual beneficial effects of SGLT2-i on renal oxygenation were, however, unconfirmed in a recent study assessing the chronic effects of SGLT2-i on renal oxygenation and perfusion. In particular, the effects of semaglutide (a GLP-1 RA) and empagliflozin or their combination for 32 weeks were assessed by Gullaksen and colleagues. Semaglutide decreased cortical and medullary perfusion, whereas empagliflozin did not affect kidney perfusion while decreasing medullary oxygenation [39]. When both drugs were combined, medullary oxygenation and renal perfusion declined [39]. The authors concluded that the nephroprotective effects of SGLT2-i were probably not due to an improvement in medullary oxygenation [39]. However, to the best of our knowledge, kidney perfusion before and after treatment with SGLT2-i has not been thus far studied with PET, and it would be relevant to assess the acute and chronic effects of these new drugs on renal perfusion and oxidative metabolism. 

Lastly, in an elegant study, Pruijm and colleagues [34] measured cortical oxygenation in patients with CKD and in healthy controls. Patients with CKD showed decreased cortical oxygenation, and at 3 years of follow-up, those who at the baseline had a higher outer R2* or a flat R2* slope had a three-fold higher risk for renal replacement therapy or a >30% increase in serum creatinine [34].

### 1.4. Basics of PET and Renal Cortical Perfusion

To measure renal perfusion, the blood volume that passes through a unit mass of renal tissue in a given time (mL∙g^−1^∙min^−1^) must be quantified. The signal intensity changes within the abdominal aorta; therefore, an arterial input function is needed when quantitative parameters are measured. Semi-quantitative measurements of renal perfusion only require recording signal intensity changes within the kidney. Quantitative imaging methods are able to give regional renal perfusion instead of only whole-kidney perfusion [40]. 

Like ASL MRI, positron emission tomography (PET) provides a non-invasive quantitative method to study renal regional perfusion. The PET signal detection generates three-dimensional images in which each voxel provides an estimate of the radionuclide concentration within the volume of tissue represented by that voxel. There is no need for a contrast agent, and the amount of ionizing radiation is minimal. Thus, PET is a safe and promising method for studying renal perfusion in patients with CKD. 

For the PET assessment of renal cortical perfusion, a positron-emitting radiopharmaceutical that traces renal perfusion is required. PET exploits the physics of positron-electron annihilation when localizing the site of the positron decay inside the patient’s body. As a result of annihilation, a pair of photons is detected outside the patient´s body by gamma detectors [41]. Correction for decay, dead time, and photon attenuation is needed. The distribution of the positron-emitting tracer in the kidney can be reconstructed to a three-dimensional image by mathematical algorithms. Perfusion quantification after the intravenous administration of a tracer demands the application of compartmental models describing the relationship between tissue count rates and the rate of regional perfusion. The conversion of the imaged tissue count rates into absolute numeric values of perfusion also requires knowledge of the quantity of the tracer in the arterial blood. Since an arterial input function (i.e., the concentration of the radioactive tracer in the blood over time) is needed for the analysis of PET data, these data can be obtained either by frequent arterial or “arterialised” blood sampling or by “extracting” the atrial input function directly from the PET data. The latter can be obtained by drawing small regions of interest in the aorta or within the left ventricle cavity [41]. PET provides only moderate spatial resolution, and this leads to a partial volume effect that causes the overestimation or underestimation of perfusion. 

### 1.5. Tracers in Renal Perfusion PET

An optimal perfusion tracer should show complete and perfusion-rate-independent extraction from arterial blood during its first pass transit through the renal capillaries [41]. 

The most widely used tracer to assess perfusion is [^15^O]H_2_O, which is an inert and freely diffusible tracer already used in several PET-based studies to evaluate renal perfusion. One-compartment or two-compartment kinetic models have been used for [^15^O]H_2_O-PET [41]. Although in our centre, as in others, this is the most widely used tracer to assess perfusion, its short half-life (2 min) of [^15^O] necessitates a nearby cyclotron and, thus, limits its wider availability. In addition, the reversible binding and fast dynamics of the [^15^O]H_2_O tracer are technically error-prone due to the relatively low temporal resolution of PET [41].

Kidney perfusion measured with the gold standard method of para-aminohippuric acid (PAH) clearance and [^15^O]H_2_O-PET-based kidney perfusion is directly related to each other in both healthy subjects and in patients with CKD [42,43]. Moreover, renal cortical perfusion measured by [^15^O]H_2_O-PET has been shown to correlate with estimated GFR (eGFR) [44].

**[^13^N]-ammonia** (half-life of 10 min) has an extraction fraction that varies with the rate of perfusion, and it is neither freely diffusible nor inert. However, the 10 min half-life of [^13^N]-ammonia and its prolonged retention in the kidney allow extended image acquisition periods, thereby improving the data and image quality [41]. Limitations of the [^13^N]-ammonia approach include the need to correct for [^13^N]-ammonia-metabolites in the blood, and the instability of the model at both very high and very low perfusion levels, resulting in the high coefficients of variation in renal perfusion estimates [41]. A linear correlation between [^15^O]H_2_O-PET and [^13^N]-ammonia-PET-based renal cortical perfusion has been reported [45]. 

**[^11^C]-acetate** (half-life of 20 min) can assess both kidney perfusion and kidney oxygen consumption. [^11^C]-acetate- and [^15^O]H_2_O-PET-based renal cortical perfusion correlate with each other [46].

**[^82^Rb]-Chloride** (half-life of 75 s) has also been used to assess renal perfusion, with some studies suggesting that [^82^Rb]-chloride-PET can measure effective renal plasma flow [47].

The PET studies that have assessed renal cortical perfusion are presented in Table 1.

### 1.6. Clinical Settings Where Renal Perfusion Has Been Assessed with PET

In healthy subjects, the values of renal cortical perfusion measured with [^15^O]H_2_O-PET have ranged from 1.6 mL∙g^−1^∙min^−1^ to 4.7 mL∙g^−1^∙min^−1^ (Table 1). [^15^O]H_2_O-PET studies have shown reduced renal cortex perfusion in patients with CKD compared to healthy subjects [43,48] and decreasing values through CKD stages (2.2 mL∙g^−1^∙min^−1^ in CKD stage 3 [42], 2.1 mL∙g^−1^∙min^−1^ in CKD stage 3–4 [42], and 1.3 mL∙g^−1^∙min^−1^ in CKD 4–5) [48]. 

In the only [^15^O]H_2_O-PET study performed thus far in kidney transplant recipients (N = 19, median time from transplantation 33 months; eGFR 55mL/min), renal cortical perfusion was compared to healthy controls [44]. No statistically significant difference emerged in renal cortical perfusion between the two groups [2.2 (2.0–3.0) mL∙g^−1^∙min^−1^ vs. 2.2 (2.4–4.0) mL∙g^−1^∙min^−1^ for patients and healthy controls, respectively, with the median (IQR)]; however, kidney transplant recipients had higher renal vascular resistance (mean arterial pressure/renal cortical perfusion) compared to the controls [44]. 

Three studies have evaluated renal cortical perfusion in patients with other diseases than CKD. In the study of Assersen et al., there was no difference in the renal cortical perfusion values measured by [^15^O]H_2_O-PET between patients with hypertension and no CKD and healthy controls [49]. In patients with morbid obesity, when compared to healthy lean subjects, there were no differences in cortical or medullary perfusion per unit of tissue mass (mL/min/100 g of tissue). However, when accounting for kidney volume, obese individuals received a higher total renal blood flow (mL/min) compared to lean controls [50]. In that study, patients who underwent bariatric surgery and renal perfusion measurements repeated after major weight loss showed a significant reduction in the total renal blood flow [50]. Lastly, renal cortical perfusion has also been assessed in patients with heart failure: such patients receive a lower blood flow to the renal cortex compared to healthy controls [51].

**Table 1:** Renal cortical perfusion in PET studies.

**Reference**	**N and Type of Subjects**	**Tracer**	**Cortex Perfusion** **(mL·g^−1^·min^−1^)**
Nitzsche et al., 1993 [45]	20 healthy subjects	[^15^O]H_2_O [^13^N]-ammonia	4.7 (0.3) 4.6 (0.5)
Middlekauff et al., 2001 [51]	29 healthy subjects	[^15^O]H_2_O	4.4 (0.1)
Middlekauff et al., 1997 [52]	19 healthy subjects 19 pts with heart failure	[^15^O]H_2_O [^15^O]H_2_O	4.2 (0.1) 3.0 (0.1)
Alpert et al., 2002 [43]	5 healthy subjects 10 pts with renal disease	[^15^O]H_2_O [^15^O]H_2_O	3.4 (0.4) 2.1 (1.1)
Juillard et al., 2002 [42]	8 pts with CKD	[^15^O]H_2_O	2.2 (2.0)
Kudomi et al., 2009 [53]	6 healthy subjects	[^15^O]H_2_O	1.6 (0.6) * 3.6 (2.2)
Damkjær et al., 2010 [54]	9 healthy subjects	[^15^O]H_2_O	4.7 (0.3)
Damkjær et al., 2012 [55]	7 healthy subjects	[^15^O]H_2_O	3.6 (0.1)
Assersen et al., 2019 [49]	12 healthy subjects 6 pts with hypertension	[^15^O]H_2_O	4.1 (0.3)
Rebelos et al., 2019 [50]	15 healthy subjects 23 women with obesity	[^15^O]H_2_O	2.7 (104) 2.6 (62)
Koivuviita et al., 2012 [48]	10 healthy subjects 7 pts with CKD 17 pts with RAS and CKD	[^15^O]H_2_O	1.8 (0.3) 1.26 (0.5) 1.43 (0.4)
Päivärinta et al., 2019 [44]	10 healthy subjects 19 pts with kidney Tx	[^15^O]H_2_O	2.7 (2.4–4.0) 2.2 (2.0–3.0)
Normand et al., 2019 [46]	10 healthy subjects	[^15^O]H_2_O [^11^C]-acetate	3.3 (0.7) 1.7 (0.3)

N, number of subjects. Data are mean (SD), median [IQR], median (Q2–Q3). *, renal cortical perfusion was measured based on K1 and K2, respectively. RAS, renal artery stenosis, CKD, chronic kidney disease.

### 1.7. Reproducibility of Renal Cortical PET Perfusion

Nitzsche et al. performed repeat measurements of renal blood perfusion using [^13^N]-ammonia PET and reported that the individual interstudy difference was 5.0% [45]. The individual interstudy difference in [^15^O]H_2_O measurements was even smaller, ~2.2%. The interstudy differences of the renal cortical blood perfusion estimates within each individual approach were insignificant [45]. In the study of Normand et al., the typical errors were 0.36 and 0.22 for [^15^O]H_2_O and [^11^C]-acetate and the interclass correlation coefficient, which were 0.64 and 0.26, respectively [46].

### 1.8. Assessment of Renal Metabolism Using PET

The kidney is a key metabolic player, with the ability to both produce and dispose of glucose. Gluconeogenesis takes place in the cortex and, in particular, at the proximal tubular cells (PCT), which are the only renal cells characterised by glucose-6-phosphatase activity [56]. Glucose disposal occurs both via glucose elimination in the urine and through glucose uptake and utilisation by renal cells. Cells in the cortical region use glucose and other substrates, whereas cells in the medulla predominantly rely on glucose [57]. Renal substrate uptake rates have been assessed using the arterial-venous difference technique. Currently, this invasive method is seldom used in specialised centres and has the drawback of yielding whole-organ substrate uptake values rather than differentiating between cortical and medullary substrate uptake rates. Only recently, PET has been introduced to assess renal metabolism. 

We have previously shown that when using 14(R,S)-[^18^F]Fluoro-6-thia-heptadecanoic acid ([^18^F]FTHA) PET to study renal fatty acid uptake (FFA), the kidney (cortical and medullary) FFA uptake is higher in obese individuals compared to healthy lean controls. These patients were re-studied six months after bariatric surgery, and we observed that, despite significant weight loss and an improvement in insulin sensitivity, renal FFA uptake remained high. This might be attributed to the ongoing catabolic state and increased FFA availability [50].

**Figure 2:**
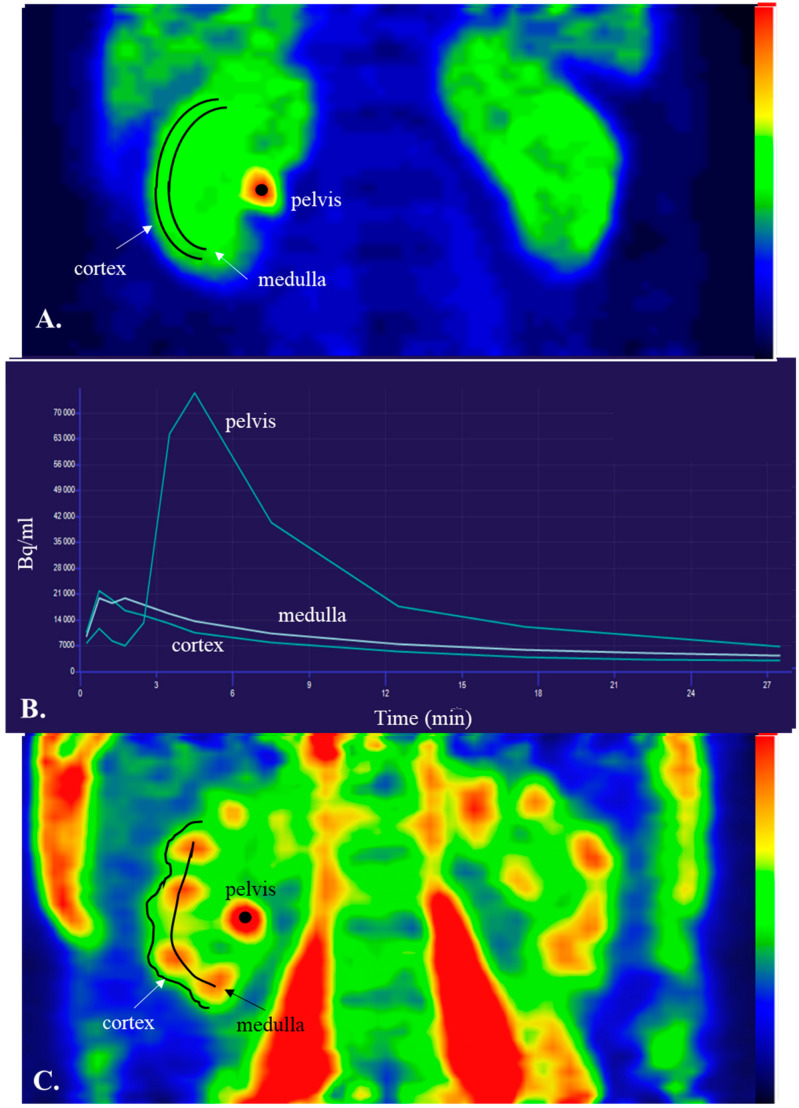
(**A**,**B**) Show an “early” scan immediately after [^18^F]FDG injection and the time–activity curves in the renal cortex, medulla and pelvis. Notice that [^18^F]FDG quickly reaches the renal pelvis (Figure 1b). (**C**) Shows a “late” scan acquired ~60 min after [^18^F]FDG injection. Data from the same subject are shown.

We have also recently used [^18^F]FDG-PET to assess regional kidney glucose metabolism, which has never been measured before [58]. The main problem with this approach is that [^18^F]FDG is excreted in the urine while, in the presence of normoglycaemia, virtually all the filtered glucose is re-absorbed by SGLTs; consequently, the [^18^F]FDG radioactivity remaining within the renal tubular space is mistakenly “read” as part of the glucose uptake by the surrounding cells. To tackle this problem, we acquired renal radioactivity data as late scans (~90 min from [^18^F]FDG injection) to allow for most of [^18^F]FDG excreted in the urine to be already washed out of the kidneys toward the bladder. Then, using the amount of [^18^F]FDG excreted in the urine at the end of the PET studies, it was possible to calculate the remaining intratubular [^18^F]FDG activity and correct the results. Through this approach, we showed that lean healthy controls had higher cortical and medullary GU rates compared to patients with obesity under both fasting and insulin clamp conditions (1 mU/kg/min). Moreover, we found that cortical, but not medullary, GU was enhanced during insulin clamp compared to the fasting study. Taken together, these data suggest that the renal cortex is an insulin-sensitive tissue [58]. Moreover, this study demonstrated that, albeit the fact that [^18^F]FDG-PET results underestimate renal glucose uptake, this method can still yield clinically useful information regarding renal metabolism [58]. Representative images of an [^18^F]FDG scan during the euglycemic hyperinsulinemic clamp are shown in Figure 2.

## 2. Discussion

Compared to the time-honored gold standard method for assessing renal perfusion (i.e., PAH clearance), the use of imaging to assess renal perfusion provides several substantial benefits. First, imaging is less invasive (in the case of fMRI, only images are acquired, whereas in the case of PET, the most widely used tracer [^15^O]H_2_O only requires the i.v. administration of the tracer, but no blood sampling). Acquisition times are also much shorter (~5 min for an [^15^O]H_2_O, and ~10 min for ASL MRI [30]) compared to a standard 2 h study that is necessary for the PAH clearance method. Importantly, imaging allows for the independent evaluation of the renal perfusion of the two kidney regions characterised by major differences in perfusion, whereas PAH only yields estimates of the total renal perfusion. The latter benefit of imaging also holds true when comparing invasive methods, such as the AV differences technique, to evaluate oxygen or substrate extraction by the kidneys, whereas PET can differentiate between the uptake rates in the cortex and the medulla, though not perfectly, as described below.

**Figure 3:**
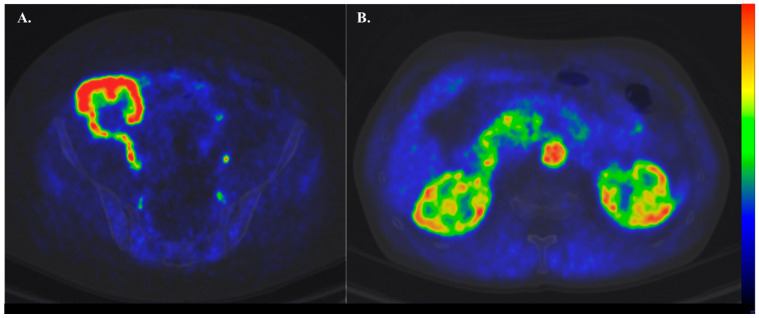
Representative [^15^O]H_2_O-PET images of a kidney transplant patient (**A**) and a healthy lean control (**B**).

Only a few studies have used these potent imaging modalities to assess renal physiology, and most of these studies have been performed in healthy subjects. Thus far, [^15^O]H_2_O is the most widely used PET tracer to assess renal perfusion (Figure 3). Overall, renal cortical perfusion measured by [^15^O]H_2_O PET has given consistent results across studies; most importantly, such studies have confirmed earlier findings using PAH clearance. It is also important to underline that even though [^15^O]H_2_O is the most widely used tracer to assess renal perfusion, the involved modelling is still suboptimal, and efforts are being made to improve it. [^13^N]-ammonia also seems to be a reliable tracer for imaging renal perfusion, although there is the need to correct for [^13^N]-ammonia metabolites in the blood, and the [^13^N]-ammonia model is unstable at both very high and very low perfusion levels. [^11^C]-acetate is a promising tracer because it can provide not only information regarding perfusion but also give information regarding oxidation. All these PET tracers show relatively short half-lives, thus confining their main use in PET centres with cyclotrons. 

Still, there are some further methodological aspects to consider when using PET to assess both renal perfusion and renal substrate uptake rates. First, the renal cortex and renal medulla are very close areas, and due to the relatively poor spatial resolution of PET, it is not possible to completely differentiate the signal between these two structures. Apart from the spatial resolution, drawing an accurate region of interest might also be biased as it is impossible to define the border between the cortex and medulla without the use of contrast-enhanced CT or MRI. Few studies have attempted to exactly define this border. Contrast-enhanced CT was used in two studies with [^15^O]H_2_O [49,55]. In the only [^11^C]-acetate study of renal cortical perfusion, T1 weighted MRI was used to define the border between the cortex and medulla [46]. Damkjær et al. introduced a new technique to differentiate between the renal cortex and medulla: tissue layers with a thickness of one voxel were eliminated stepwise from the external surface of the volume of interest (voxel peeling) until there was no decrease in the blood perfusion and the medulla was reached [54,55]. Renal PET/MRI studies have the additional advantage of better anatomical distinction between these two regions compared to PET/CT studies and also of a lower radiation burden to the studied subjects [59].

The most novel fMRI modalities (ASL and BOLD) have also started to be used in specialised centres, with some of the latest publications focusing on the hemodynamic effects of anti-hyperglycemic agents with nephroprotective effects.

Even though BOLD fMRI is, in fact, the only non-invasive way that is currently available to assess renal oxygenation in humans, it should be kept in mind that it is not a direct measure of tissue oxygenation, as changes in the blood volume can affect the amount of deoxyhaemoglobin in the tissue. Recently, a pilot study in healthy subjects and in patients with CKD addressed this issue by combining BOLD fMRI with ferumoxytol to estimate the fractional renal tissue blood volume. With the combination of these two methods, the authors could estimate tissue oxygenation and showed that, indeed, patients with CKD had decreased StO_2_ and blood PO_2_ in the renal cortex and the renal medulla compared to the healthy subjects, confirming the chronic hypoxia hypothesis [60].

Currently, at the Turku PET Centre there is ongoing work to validate the use of [^15^O]O_2_ for the assessment of renal oxygenation with PET.

When applying functional renal imaging (PET or fMRI), it should be considered that physiologic parameters might also affect kidney perfusion results, some of which might be easy and others impossible to control. It is now well-established that, before a kidney perfusion study, salt and water intake should be balanced and reported, as salt loading is associated with renal cortical vasoconstriction and decreased cortical perfusion [49]. Indeed, recent reports on this topic used standardised hydration and salt-consuming protocols before performing the studies. However, kidney cortical perfusion has also been shown to decrease during handgrip exercises and during mental stress via the activation of the sympathetic nervous system [47,51,52]. These data suggest that study participants should be put in at-ease conditions, something that might be difficult to obtain, for instance, if a subject suffers from mild claustrophobia during the imaging session. Future studies should evaluate stress hormones to correlate with the results of kidney perfusion measurements, whereas, in paired experiments, similar study conditions should be ensured on different experimental days. 

From a physiologic standpoint, whether renal oxygenation and renal perfusion are directly and linearly interrelated is not known, mainly due to the difficulties in assessing these aspects in humans. Progress in medical imaging makes it possible to study aspects of renal physiology that in the past relied only on the measurement of AV differences. In this respect, renal imaging may give substantial new insights regarding the mechanisms of action of new potent nephroprotective drugs. Some investigations on the renal hemodynamic effects of SGLT2-i and GLP1-RA have already been published [39], and more research on this topic is expected.

It should be also pointed out that, so far, there are no studies in which renal cortical perfusion has been measured with both PET and an MRI. Ongoing research on the iBEAT project should clarify this aspect [61]. Considering the lower radiation burden needed in studies using ultralong field-of-view PET scanners, a combination of PET with MRI in future investigations is expected to increase our insight into the physiology of renal function and the pathophysiology of kidney diseases. Apart from the methodological considerations described above, the high costs of these imaging modalities and the need for the “on-site” production of short-lived radiotracers are important aspects that have thus far limited a broader use of renal imaging.

Our research group has been a pioneer in evaluating renal substrate uptake rates using PET. With this method, we have shown that, in the context of insulin resistance, renal FFA uptake is increased, whereas renal glucose uptake during standardised insulinisation is decreased. Future reports might further elaborate on whether an altered renal substrate uptake is associated with progressive kidney damage and whether a lifestyle modification or bariatric surgery could correct it [62], evaluating the interplay between renal metabolism and the adjacent organs, such as renal sinus fat [63], and the liver [64]. With respect to the liver, recent reports have shown that carriers of the PNPLA3 p.I148M variant (i.e., the main genetic determinant of non-alcoholic fatty liver disease) have impaired kidney function [65]; however, the mechanisms linking hepatic steatosis with a worse renal function are still partially unknown. 

In conclusion, renal function imaging “is here to stay” and may provide useful insight into the physiology (perfusion, oxygenation, metabolism) of the kidney; our ultimate goal is to protect these organs from the various insults metabolic diseases and modern lifestyles impose on them. 

## Data Availability

Not applicable.
